# First person – Monika J. Tomecka

**DOI:** 10.1242/dmm.041848

**Published:** 2019-09-01

## Abstract

First Person is a series of interviews with the first authors of a selection of papers published in Disease Models & Mechanisms (DMM), helping early-career researchers promote themselves alongside their papers. Monika Tomecka is first author on ‘Clinical pathologies of bone fracture modelled in zebrafish’, published in DMM. Monika conducted the research described in this article while a PhD student in Tom Carney's lab at the Institute of Molecular and Cell Biology (IMCB), A*STAR (Agency for Science, Technology and Research), Singapore and Henry Roehl's lab, Department of Biomedical Science, The University of Sheffield, UK. She is now a CEO at uFraction8, an engineering company that develops biotech instruments for industrial cell culture harvesting.


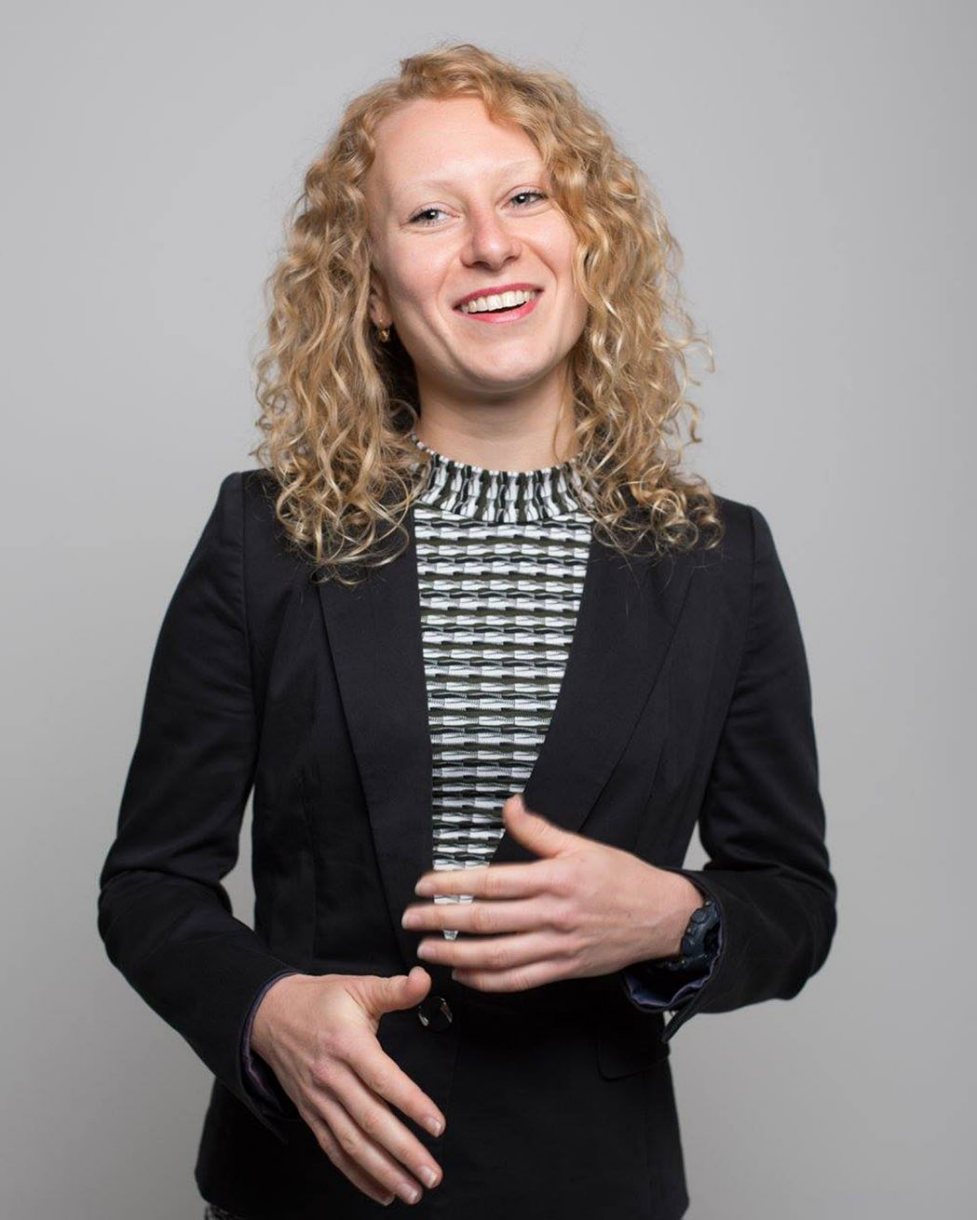


**Monika Tomecka**

**How would you explain the main findings of your paper to non-scientific family and friends?**

Bone fracture injuries, as well as their consequences, are important clinical issues. Several questions raised about them cannot be answered from observations of patients; therefore, animal models of human bone fractures are needed. I have developed zebrafish crush injury as an accurate model for human bone fracture repair. I established the model and further evaluated its usage in the zebrafish bone mutant *frf*, a known model for osteogenesis imperfecta (OI; also known as brittle bone disease). People with this disease have extremely fragile bones and tend to break them very easily. I tested common human bone disease drugs – bisphosphonates – on the zebrafish crush model, further showing the usefulness and relevance of the model. Upon treatment, I observed a significant impact on fracture healing, as well as on spontaneous fracture formation in juvenile OI zebrafish. Last, but not least, I decided to examine the impact of infection on fracture healing. I introduced controlled infections to the crush site and found that persistent infection inhibits fracture repair. All these results mimic human clinical data. In conclusion, I established a zebrafish bone crush model relevant to human physiology and pathology. I proved its usefulness in bone fracture healing characterisation studies, in determining bone pathogenicity as well as in bone-related drug treatment efficiency, and infection disturbance in bone repair.

“The ability to recreate precise genetic lesions offers the ability to provide personalised models for genetic bone disorders of human patients in the near future.”

**What are the potential implications of these results for your field of research?**

Whilst studies of amputation of the adult zebrafish tail attracted much interest as a model for regeneration, there has been very limited analysis of the process of repair of bone fractures. Zebrafish crush injury, shown in my paper, is an accurate model for human bone fracture repair and is an enabling tool for human fracture research in fish. To date, the majority of fracture studies were performed in bigger animals such as rodents and dogs. Zebrafish offer a number of advantages as a model system, including ease of imaging and genome modifications, allowing fast generation of bone-specific transgenic lines and mutants. The ability to recreate precise genetic lesions offers the ability to provide personalised models for genetic bone disorders of human patients in the near future. Use of the crush procedure developed here adds to the methods for characterising the ensuing bone phenotype, allowing direct evaluation of the outcome of the genetic lesion on fracture repair. Furthermore, the fertility of zebrafish provides high sample numbers for experimental replicates, whilst compounds are easy to administer through immersion.

“It was amazing to find out that zebrafish tail bones can actually be a great model for human bones research.”

**What has surprised you the most while conducting your research?**

It was amazing to find out that zebrafish tail bones can actually be a great model for human bones research. It was surprising and amazing to see that most of the results of crush experiments mimicked human clinical data. I have often dealt with disbelief from my peers that ‘fish bones’ can be a good representation of human bones. I was positively surprised when I found and proved that they indeed can.

**What changes do you think could improve the professional lives of early-career scientists?**

I truly believe that early-career scientists should focus not only on their studies but also on their personal development, trying to gain new skills and experiences. I think that changes could involve increasing the amount of such opportunities. I am aware that there are already several programmes, workshops, personal development conferences etc., but sometimes their reach is limited or scientists are not convinced about their usefulness. I think we should induce a change of mindset both in early-career scientists and their PIs. Catching opportunities, looking for interdisciplinary solutions and collaborating with industry are at the top of my advice list.

“I truly believe that early-career scientists should focus not only on their studies but also on their personal development, trying to gain new skills and experiences.”

**What's next for you?**

For now, I have decided to move on from the laboratory and become an entrepreneur. I have founded a successful biotech company, uFraction8, and I am continuing to spread my love for science in my own, entrepreneurial way. I refer to myself as an entrepreneur scientist. In building my business I use skills and knowledge collected through my studies and the PhD research. On the other hand, because I'm creating biotech hardware technology, which involves a lot of engineering, I am learning new things every single day. I have not had much previous experience in finance and running a business either so, naturally, those are now the skill-set gaps that I am currently working on. I have always been a chatty scientist, and now I spread my scientific and business knowledge through talks, presentations and keynote speeches, which makes me feel like a ‘fish in the water’. I keep attending scientific conferences and interact with researchers to keep up-to-date in the field and try to look for interdisciplinary solutions to scientific problems. I am always looking for collaborators and people willing to combine our potentials to create something incredible.
